# Potential advantage of magnetic resonance imaging in detecting thoracic wall infiltration in pleural mesothelioma: A retrospective single-center analysis

**DOI:** 10.1016/j.xjon.2024.10.012

**Published:** 2024-10-22

**Authors:** Isabel Barreto, Sabine Franckenberg, Thomas Frauenfelder, Isabelle Opitz, Olivia Lauk

**Affiliations:** a Department of Thoracic Surgery, University Hospital Zurich, Zurich, Switzerland; b Institute of Diagnostic and Interventional Radiology, University Hospital Zurich, Zurich, Switzerland

**Keywords:** extension of resection, patient selection for surgery, pleural mesothelioma, preoperative MRI, restaging

## Abstract

**Objectives:**

Thoracic wall infiltration in pleural mesothelioma determines the extent of resection and can be an important prognostic factor. Currently, standardized imaging for restaging after neoadjuvant systemic therapy comprises contrast-enhanced computed tomography or positron emission tomography. Additional thoracic magnetic resonance imaging could better discriminate chest wall infiltration preoperatively and increase staging accuracy. For this reason, the added benefit of magnetic resonance imaging was evaluated at our center.

**Methods:**

A retrospective analysis of the extended imaging protocol was performed from July 2018 to March 2024, including a descriptive analysis for the patient's sex, age, tobacco consumption, asbestos exposure, histological subtype, TNM stage, Modified Response Evaluation Criteria for Solid Tumors in solid tumors, and number of neoadjuvant therapy cycles. Preoperative restaging included routine imaging and magnetic resonance imaging. After histological diagnosis of pleural mesothelioma, neoadjuvant therapy was conducted, followed by intended macroscopic complete resection, with intraoperative biopsies of suspicious chest wall lesions. Computed tomography and magnetic resonance imaging results were compared with intraoperative biopsies.

**Results:**

Twenty-six patients (mean age, 65.50 years, 11.50% female) with operable pleural mesothelioma were included. Of the 11 patients with histologically proven chest wall infiltration, 10 (90.91%) had a cT-stage 3 or greater and 4 (36.36%) underwent surgery that resulted in an R2 resection. Thoracic magnetic resonance imaging showed a high sensitivity (90.91%) for the detection of chest wall infiltration, especially when compared with the computed tomography scan (9.09%).

**Conclusions:**

With the adjunctive use of magnetic resonance imaging, we demonstrated a higher sensitivity for detection of chest wall infiltration compared with conventional imaging before surgery. This may improve patient selection for surgery. Nevertheless, larger studies are required to confirm these results.

## Graphical Abstract

**Figure:**
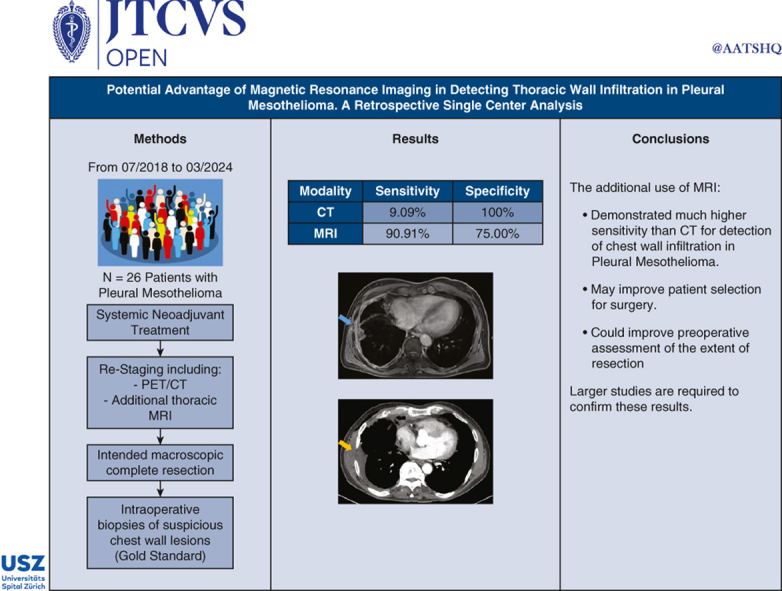

**Figure:**
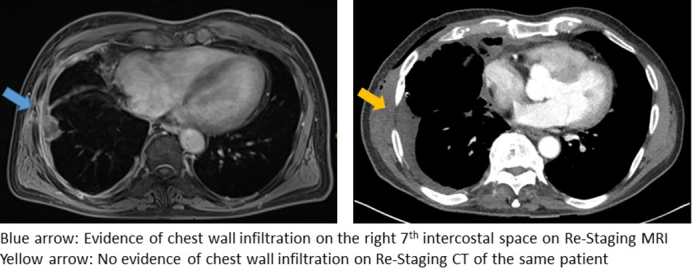
Example of better detection of mesothelioma thoracic wall infiltration on MRI.

**Central Message**

Additional thoracic MRI shows a higher sensitivity than contrast-enhanced CT for the detection of chest wall infiltration of PM.

**Perspective**

The additional use of thoracic MRI during restaging in PM showed a higher sensitivity for the detection of chest wall infiltration than standard imaging. Consequently, this new protocol could allow for a better preoperative planning of the extent of the resection, as well as improved patient selection for complete macroscopic resection, possibly improving outcomes and survival.

See Commentator Discussion on page 326.

Pleural mesothelioma (PM) is an aggressive primary malignancy of the pleura that remains a clinical challenge, with the 5-year survival continuing to languish at 5% to 10% despite treatment.^1^ According to current guidelines, patients are treated within a multimodal therapy approach, including macroscopic complete resection (MCR) with preoperative or postoperative systemic therapy.^2^, ^3^, ^4^

Thoracic wall infiltration is considered a sign of advanced disease burden, and because it determines the extent of the resection, it is an important prognostic factor.^5^ However, it also presents a radiologic challenge, being in some cases first diagnosed intraoperatively. To date, standard imaging for restaging after neoadjuvant systemic therapy in patients with PM is contrast-enhanced computed tomography (CT) or positron emission tomography (PET).^6^^,^^7^ Thoracic magnetic resonance imaging (MRI) in patients with PM has been shown to be a useful tool not only for performing precise tumor volume measurements^8^ but also for better discrimination of chest wall infiltration.^9^^,^^10^ To test this hypothesis and better detect chest wall infiltration before MCR of PM, we introduced an extended protocol at our center, which included standard imaging and additional MRI after induction chemotherapy to restage before surgery.

## Material and Methods

### Study Design

**Figure 1:**
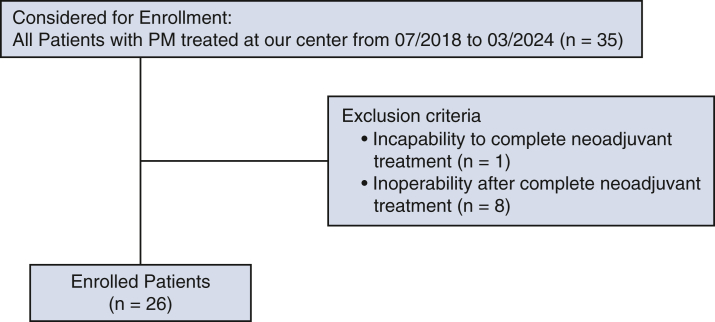
CONSORT diagram regarding patient selection. *PM*, Pleural mesothelioma.

A retrospective analysis of the new MRI protocol for patients with PM was performed from July 2018 to March 2024. Of 35 patients with PM treated at our center during the observation period, 26 with resectable disease were included (Figure 1). Exclusion criteria included incapability to complete neoadjuvant therapy (1 patient) and inoperability after complete neoadjuvant treatment (8 patients). All included patients gave their written informed consent for publication. The study protocol was approved by the Ethics Committee of Human Subjects Protection of Zurich (February 9, 2021), and received the approval Number 2020-02566.

All patients had mediastinal staging by endobronchial ultrasound (EBUS) or mediastinoscopy according to the lymph node size on imaging and availability.

Mediastinoscopy was always performed within the same operation with diagnostic thoracoscopy for overall staging and to exclude contralateral lymph node disease. If fresh-frozen section revealed suspicion of PM, mediastinoscopy was additionally performed. In case of histological nodal disease at diagnosis, PET-CT was mandatory after neoadjuvant treatment. In case of PET-positive lymph nodes, which was never the case in our cohort, mediastinoscopy/EBUS would have been performed for restaging purposes. Preoperative N1 or N2 disease was no contraindication for surgery. Contralateral lymph node disease precluded surgery.^11^

The following parameters were extracted from the patient files: patient's sex, age, nicotine consumption, asbestos exposure, histological subtype, TNM stage, Modified Response Evaluation Criteria for Solid Tumors (mRECIST), systemic therapy regimen, and number of neoadjuvant therapy cycles.

After histological diagnosis of PM, 3 to 6 cycles of neoadjuvant therapy were conducted, consisting of chemotherapy alone (cisplatin/carboplatin and pemetrexed) or with bevacizumab (in 14 cases). Three to 5 weeks after the last cycle, thoracic MRI was performed, followed 1 to 16 days later by intended MCR, with intraoperative biopsies of suspicious chest wall lesions.

All included patients received complete neoadjuvant therapy and had a tumor deemed resectable. Preoperative restaging included routine imaging (CT or PET/CT) and additional MRI.

Thoracic MRI was conducted using the MAGNETOM Skyra system from Siemens Healthineers at a field strength of 3 Tesla. A specialized MRI protocol was developed, based on the consensus statement from the International Mesothelioma Interest Group.^6^ This protocol included standard T1-and T2-weighted sequences (T1 VIBE Dixon, T2 BLADE, T2 BLADE with fat suppression) to differentiate MPM from the chest wall, diaphragm, and pericardium. Postcontrast T1-weighted sequences (Dotarem; immediately after injection and at 2, 3, 4, and 6 minutes) provided enhanced visualization of the extent and potential invasion of PM.^12^ Additionally, diffusion-weighted imaging was included (2-dimensional echo-planar imaging sequence for diffusion-weighted imaging with fat suppression) with 2 different diffusion sensitivities (b-values of 100 and 1500) to provide information on tissue diffusion characteristics. This can be helpful in assessing tissue composition and treatment response.^6^^,^^13^^,^^14^ The image data were evaluated on a PACS workstation. All MRI images were interpreted by a dedicated team of thoracic radiologists and reviewed by the head of the team, a senior consultant with more than 20 years of experience in chest radiology.

Patients undergoing surgery had a maximum of 3 chest wall infiltration sites on preoperative imaging, although there was no official cutoff for surgery. Depending on tumor volume before chemotherapy, histological subtype, C-reactive protein before chemotherapy, and tumor progression after chemotherapy, the feasibility and indication of MCR were reevaluated according to the Multimodality Prognostic Score.^15^

During surgery, importance was given to repeated intraoperative biopsies and frozen section of the chest wall, diaphragm, and pericardium to ensure accurate intraoperative staging and to adapt the surgical procedure in case of multifocal infiltration. An extrapleural dissection of the parietal pleura was conducted by separating the pleura from the chest wall, pericardium, and diaphragm. The primary goal was to preserve the integrity of both the diaphragm and the pericardium; however, resection of these structures was performed if transdiaphragmatic or pericardial infiltration was confirmed intraoperatively by fresh-frozen sections. In a second step, visceral pleurectomy was carried out. In case of macroscopical or histological confirmed lung or visceral pleura infiltrations (also by fresh-frozen section), the visceral pleura of each lobe as well as interfissural was removed/dissected. During this process, the pleura was carefully peeled away while the patient was under positive pressure ventilation, which aids in maintaining lung expansion and ensuring a clear dissection plane. Intraoperative invasion of the intercostal muscle or any structure beyond the endothoracic fascia was defined as chest wall invasion. Once determined intraoperatively, chest wall invasion only precluded further continuation of the resection if present in more than 3 sites. Otherwise, these 3 or fewer lesions were excised with clear margins and clipped for postoperative radiotherapy. Patients with inoperable disease during surgery received partial pleurectomy.^16^

**Figure 2:**
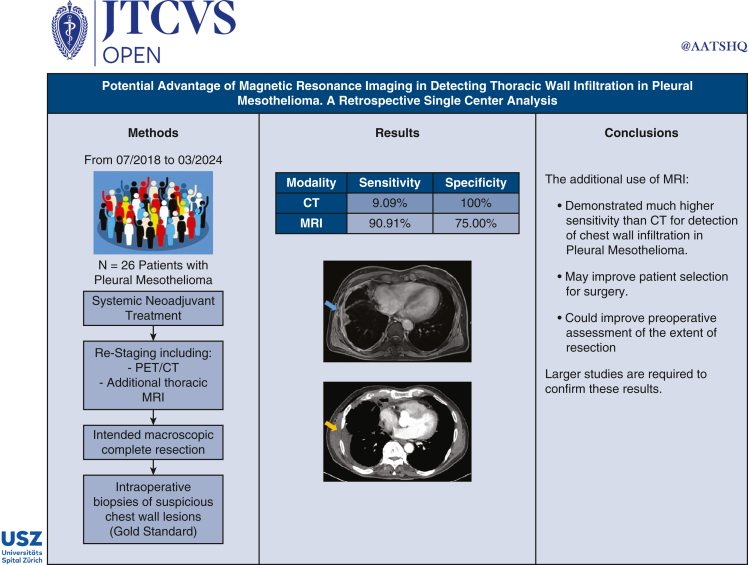
Graphical Abstract: Single-center retrospective analysis of the potential benefit of additional thoracic MRI for assessing chest wall infiltration during pleural mesothelioma restaging after neoadjuvant systemic therapy and before resection. *PET*, Positron emission tomography; *CT*, computed tomography; *MRI*, magnetic resonance imaging.

CT/MRI results were compared with the intraoperative biopsies. Sensitivity and specificity of both modalities were analyzed, and the localization of the metastatic lesions in imaging was compared with the intraoperative location. Figure 2 shows a Graphical Abstract of the study.

### Statistical Analysis

A descriptive analysis of a patient's sex, age, tobacco consumption, asbestos exposure, histological subtype, TNM stage, mRECIST, systemic therapy regimen, and number of neoadjuvant therapy cycles was performed. A subgroup analysis regarding the histologic presence or absence of chest wall invasion in the intraoperative biopsies was also carried out.

Finally, CT and MRI results were compared with the intraoperative histologic findings. Sensitivity and specificity of both modalities were calculated for all patients for extended pleurectomy and decortication and partial pleurectomy. Furthermore, the intraoperative location of the lesions was compared with the reported location on imaging.

## Results

### Demographic and Histopathological Characteristics

Mean age at diagnosis was 65.50 years (range, 45-79). Only 11.50% of the patients were female. A total of 96% of the patients had confirmed or possible asbestos exposure, and 38.46% were current or former smokers. The lymph node staging was performed by EBUS (57.69%) or mediastinoscopy (42.31%). Histological subtype was mainly epithelioid (84%). The response to the induction therapy was mostly favorable according to the mRECIST, with stable disease being reported in 46% and partial response in 50% of cases.

The 11 patients with intraoperative histologic chest wall infiltration were older at diagnosis (mean age 67.7 years) when compared with the patients with no evidence of chest wall infiltration (mean age 63.9 years). A similar male predominance (90.90% vs 86.67%) was observed in both groups.

In the subgroup with histological chest wall infiltration, more than half of cases (54.55%) had no lymph node involvement; 90.91% of these patients had a cT stage of 3 or higher. Three patients with 3 chest wall invasion sites on preoperative imaging were found to have multilevel chest wall disease intraoperatively and ultimately received a partial pleurectomy. These were 3 cases of young and otherwise healthy patients who had borderline resectable disease preoperatively, because they were found to have 3 chest wall invasion sites and tumor volumes slightly less than 500 cm³ on imaging. Despite these findings, they still met the criteria for resectability. Given their young age, an attempt was made to perform MCR. In our experience, and due to the heterogeneity of this tumor and its presentation on imaging, there are cases where imaging does not accurately reflect intraoperative findings concerning resectability. Up to 3 chest wall infiltration sites, if the remainder of the tumor is well resectable, are acceptable. As a result, these patients were taken to the operating room for exploratory thoracotomy with evaluation of the disease extent. Ultimately, due to the T4 stage, they underwent partial pleurectomy.

R2 occurred in 4 of the 11 patients with chest wall invasion (36.36%). However, only the 3 exceptional cases mentioned above (27.27%) were due to multilevel chest wall invasion. The other R2 case was due to higher tumor load.

The mean overall survival of all patients was 20.27 months and balanced between groups, although the subgroup with histological chest wall infiltration had a slightly lower mean overall survival of 19.55 months. Patient demographic and histopathological characteristics are listed in Table 1.

**Table 1:** Demographic and clinical characteristics of the included patient population

Parameter	All patients (n = 26)	Patients with intraoperative chest wall infiltration (n = 11)	Patients without intraoperative chest wall infiltration (n = 15)
Mean age, y	65.50	67.70	63.90
Sex			
Male	23 (88.46%)	10 (90.90%)	13 (86.67%)
Female	3 (11.54%)	1 (9.10%)	2 (13.33%)
Asbestos exposure			
Yes	19 (73.08%)	7 (63.63%)	12 (80.00%)
Possible	6 (23.08%)	3 (27.27%)	3 (20.00%)
No	1 (3.84%)	1 (9.09%)	0 (0.00%)
Smoking status			
Never	16 (61.54%)	5 (45.45%)	11 (73.33%)
Former	9 (34.62%)	6 (54.55%)	3 (20.00%)
Current	1 (3.84%)	0 (0.00%)	1 (6.67%)
cT-stage			
T1	8 (30.77%)	1 (9.09%)	7 (46.67%)
T2	0 (0.00%)	0 (0.00%)	0 (0.00%)
T3	16 (61.5%)	9 (81.82%)	7 (46.67%)
T4	2 (7.69%)	1 (9.09%)	1 (6.66%)
pT-stage			
T1	4 (15.38%)	0 (0.00%)	4 (26.67%)
T2	5 (19.23%)	0 (0.00%)	5 (33.33%)
T3	14 (53.85%)	8 (72.73%)	6 (40.00%)
T4	3 (11.54%)	3 (27.27%)	0 (0.00%)
pN-stage			
N0	16 (61.54%)	6 (54.55%)	10 (66.66%)
N1	8 (30.77%)	4 (36.36%)	4 (26.67%)
N2	2 (7.69%)	1 (9.09%)	1 (6.67%)
Histologic type			
Epithelioid	22 (84.62%)	10 (90.91%)	12 (80.00%)
Biphasic	3 (11.54%)	1 (9.09%)	2 (13.33%)
Sarcomatoid	1 (3.84%)	0 (0.00%)	1 (6.67%)
Staging			
Mediastinoscopy	11 (42.31%)	7 (63.64%)	4 (26.67%)
EBUS	15 (57.69%)	4 (36.36%)	11 (73.33%)
No. of induction therapy cycles (cisplatin or carboplatin, pemetrexed ± bevacizumab)			
3	14 (53.85%)	7 (63.64%)	7 (46.67%)
4	10 (38.46%)	3 (27.27%)	7 (46.67%)
6	2 (7.69%)	1 (9.09%)	1 (6.67%)
Bevacizumab	14 (53.85%)	6 (54.55%)	8 (53.33%)
Mean number of week between thelast chemotherapy cycle and MRI	4.28	4.91	3.80
mRECIST response			
Stable disease	12 (46.15%)	8 (72.73%)	4 (26.67%)
Partial response	13 (50.00%)	3 (27.27%)	10 (66.66%)
Progressive disease	1 (3.85%)	0 (0.00%)	1 (6.67%)
Mean no. of days between MRI and surgery	5.85	4.82	6.60
Type of surgery			
EPD	21 (80.77%)	7 (63.64%)	14 (93.33%)
PPD	5 (19.23%)	4 (36.36%)	1 (6.67%)
Chest wall resection	11 (42.31%)	11 (100%)	0 (0.00%)
Diaphragmatic resection	17 (65.38%)	6 (54.55%)	11 (73.33%)
Pericardial resection	17 (65.38%)	6 (54.55%)	11 (73.33%)
R2-resection	5 (19.23%)	4 (36.36%)	1 (6.67%)
R2-resection due to chest wall invasion	3 (11.54%)	3 (27.27%)	0 (0.00%)
Mean overall survival	20.27	19.55	20.80

*EBUS*, Endobronchial ultrasound; *MRI*, magnetic resonance imaging; *mRECIST*, Modified Response Evaluation Criteria for Solid Tumors; *EPD*, extended pleurectomy and decortication; *PPD*, partial pleurectomy and decortication.

### Performance of Computed Tomography Scan and Magnetic Resonance Imaging Modalities

**Figure 3:**
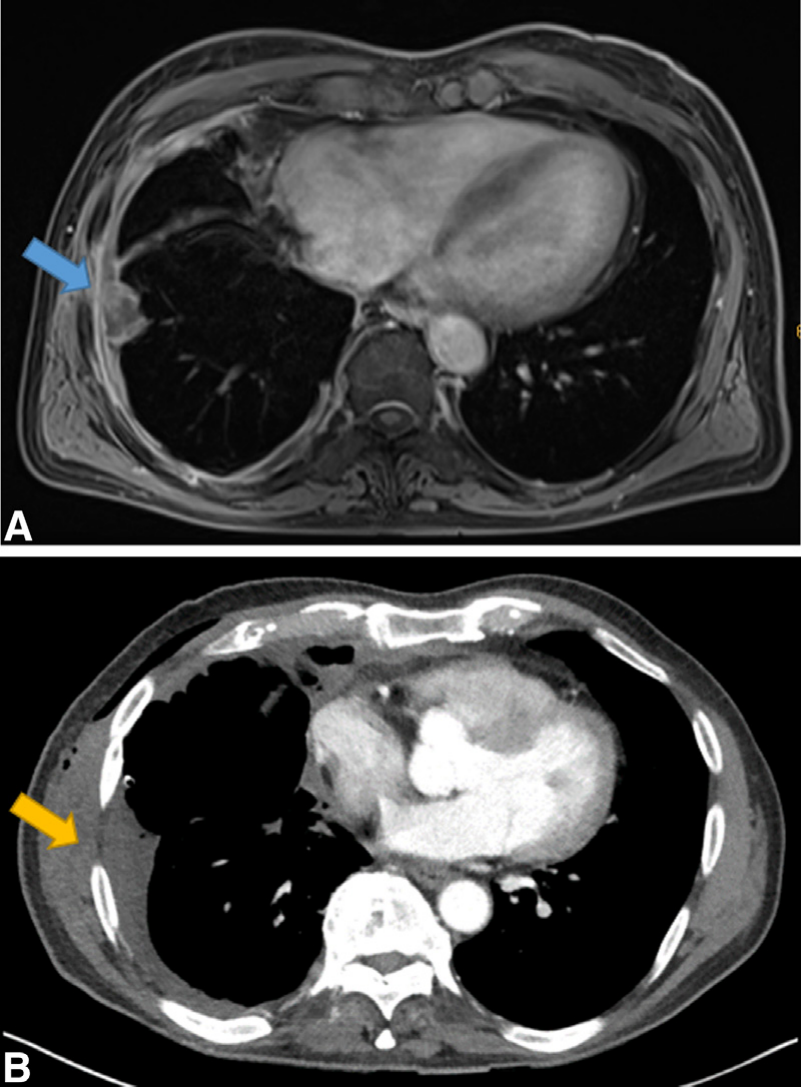
A, Thoracic MRI showing PM chest wall infiltration on the right seventh intercostal space laterally (*blue arrow*). B, Thoracic restaging CT of the same patient without evidence of PM chest wall infiltration (*yellow arrow*). Both *arrows* indicate the intraoperative site of chest wall infiltration in this case. *MRI*, Magnetic resonance imaging; *PM*, pleural mesothelioma; *CT*, computed tomography.

MRI demonstrated a higher sensitivity in detecting chest wall invasion compared with CT (90.91% vs 9.09%, real case example in Figure 3). However, the specificity of CT was 25% higher than in MRI, as outlined in Table 2.

**Table 2:** Sensitivity and specificity of computed tomography and magnetic resonance imaging

Modality	Sensitivity	Specificity	Concordance with the intraoperative location
CT	9.09%	100%	1/1
MRI	90.91%	75.00%	9/10

*CT*, Computed tomography; *MRI*, magnetic resonance imaging.

When chest wall infiltration was diagnosed in MRI, the reported location was concordant with the intraoperative location in 9 of 10 cases (Tables 2 and 3). In the only case of detection of chest wall infiltration by CT, the location on imaging was concordant with the intraoperative location.

**Table 3:** Intraoperative and magnetic resonance imaging location of chest wall infiltration

Case no.	Location of chest wall infiltration on MRI	Intraoperative location of chest wall infiltration
1	Right 5th and 7th intercostal space	Right 5th and 7th intercostal space
2	Left 6th intercostal space	Left 6th intercostal space
3	Right 6th intercostal space	Right 6th intercostal space
6	Left 3rd and 5th intercostal space	Left 3rd and 5th intercostal space
7	Right 6th intercostal space	Right 6th intercostal space
11	Right 7th intercostal space	Right 7th intercostal space
13	None (false-negative result)	Left 5th to 7th intercostal space
17	Right 7th intercostal space	Right 7th intercostal space
22	Left 6th intercostal space	Left 6th intercostal space
25	Left 8th intercostal space	Left 5th intercostal space
26	Left 2nd intercostal space	Left 2nd intercostal space

*MRI*, Magnetic resonance imaging.

A separate analysis of the sensitivity and specificity of both imaging modalities was performed according to the type of surgery (Table 4). In partial pleurectomy cases (n = 5), MRI had a particularly high sensitivity (100%) and a particularly low specificity (0%), although there was only 1 false-positive. Conversely, CT showed a particularly high specificity (100%) and a particularly low sensitivity (0%) in these cases, with 4 false-negative results. In extended pleurectomy and decortication cases (n = 21), MRI had more balanced results, with a sensitivity of 75% and a specificity of 69.23%. In this patient group, CT scan continued to exhibit an exceedingly low sensitivity (1.25%) and high specificity (100%).

**Table 4:** Sensitivity and specificity of computed tomography and magnetic resonance imaging per type of surgery performed

Modality	Sensitivity	Specificity	No. of false-positive results	No. of false-negative results
CT for EPD	1.25%	100%	0	7
MRI for EPD	75.00%	69.23%	4	2
CT for PPD	0.00%	100.00%	0	4
MRI for PPD	100%	0.00%	1	0

*CT*, Computed tomography; *EPD*, extended pleurectomy and decortication; *MRI*, magnetic resonance imaging; *PPD*, partial pleurectomy and decortication.

## Discussion

As stated in the most recent European Respiratory Society/European Society of Thoracic Surgeons/European Association for Cardio-Thoracic Surgery/European Society for Radiotherapy and Oncology guidelines, PM should be treated within a multimodal treatment including MCR if the tumor is deemed resectable and the patient considered suitable after induction chemotherapy.^11^ In North America, up-front surgery also is performed frequently as a valid alternative, according to the American Society of Clinical Oncology guidelines.^3^^,^^4^ Irrespective of the treatment sequence, preoperative patient selection represents a challenge in PM because in contrast to most other malignancies, the discrepancy in reliability between clinical and pathological staging frequently leads to unsatisfactory patient selection for radical surgery.^11^

In the case of neoadjuvant systemic therapy, restaging must follow, including assessment of treatment response by means of the mRECIST. The feasibility and indication of MCR were reevaluated according to the Multimodality Prognostic Score^15^ and the preoperative tumor volume. Specifically, Gill and colleagues showed a poorer overall survival for patients with a tumor volume greater than 500 cm^3^,^13^, ^17^ which is used as cutoff for MCR at our center.

MRI may play a complementary role in CT or PET/CT in the radiologic assessment of PM. Because of its better resolution for soft-tissue, MRI is often used to resolve equivocal findings on CT, enabling further evaluation of suspected local invasion. The primary clinical question for MRI is typically to determine the presence and extent of tumor invasion into the diaphragm, chest wall, and mediastinal structures, which aids in evaluation of resectability in preoperative planning.^9^^,^^18^^,^^19^ In this study, MRI showed a higher sensitivity than CT (90.91% vs 9.09%) regarding the preoperative diagnosis of chest wall infiltration of PM during restaging after induction therapy. Tumors of histologic epithelioid type and a pT stage of 3 or higher particularly showed a propensity for chest wall infiltration, which was not originally detected in CT, but in MRI. In cases with histological chest wall infiltration, there was an R2 resection rate of 36.36% (4 of 11 cases). However, the R2 resection rate due to multilevel chest wall invasion was only 27.27%, reflecting preoperative borderline resectable disease in fit patients who were given the chance of operative staging.

In light of these results, MRI could help us better select surgical candidates. Through larger studies using MRI, risk factors for R2 resections such as the presence/extent of chest wall infiltration^20^ and pleural thickness^21^^,^^22^ could be further studied. Defining cutoffs of these variables could allow us to better select candidates for surgery, especially when used in combination with the presence/absence of sarcopenia, a known prognostic factor.^23^ Additional use of thoracic MRI during restaging also could reduce operative times, increase accuracy of resection, and improve perioperative and postoperative outcomes and ultimately overall survival.

However, given the fact that the specificity of MRI is 25% lower than the CT scan, some caution is advisable when interpreting positive results, especially for high tumor volumes. Furthermore, an experienced thoracic radiologist should be systematically involved to reduce interobserver variability to a minimum.

### Limitations

The not entirely satisfactory specificity of MRI, especially in partial pleurectomy cases, might be due to the small sample size, because the absolute number of false-positives was not high. Moreover, the risk of false-negatives on CT was shown to be higher, regardless of disease extent, reflecting the added value of MRI in patient selection for surgery.

In any case, the small size of our cohort does not allow us to reach conclusions with high statistical power. Although a great difference in sensitivity between these imaging modalities has been observed, larger studies are needed to confirm these results.

## Conclusions

The higher sensitivity of MRI for detection of chest wall infiltration in PM might facilitate better patient selection for MCR and preoperative assessment of the extent of resection, although larger studies are required to confirm these results.

### Webcast

You can watch a Webcast of this AATS meeting presentation by going to: https://www.aats.org/resources/potential-advantage-of-magneti-7407.

**Figure:**
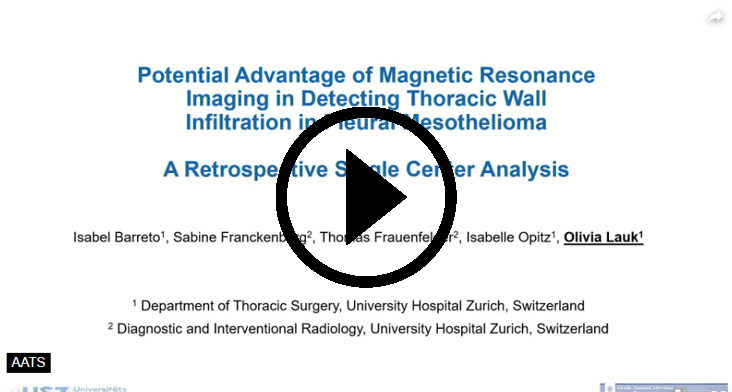

## Conflict of Interest Statement

I.O.: Roche (Institutional Grant and Speakers Fee), Roche Genentech (Steering Committee), AstraZeneca (Advisory Board and Speakers Fee), MSD (Advisory Board), BMS (Advisory Board), Medtronic (Institutional Grant and Advisory Board), Intuitive Proctorship. All other authors reported no conflicts of interest.

The *Journal* policy requires editors and reviewers to disclose conflicts of interest and to decline handling or reviewing manuscripts for which they may have a conflict of interest. The editors and reviewers of this article have no conflicts of interest.

## References

[1] Milano M.T., Zhang H. (2010). Malignant pleural mesothelioma: a population-based study of survival. J Thorac Oncol.

[2] Opitz I., Bille A., Dafni U. (2023). European epidemiology of pleural mesothelioma—real-life data from a joint analysis of the Mesoscape database of the European thoracic Oncology Platform and the European Society of Thoracic Surgery Mesothelioma Database. J Thorac Oncol.

[3] Kindler H.L., Ismaila N., Hassan R. (2018). Treatment of malignant pleural mesothelioma: American Society of Clinical Oncology Clinical Practice Guideline. J Clin Oncol.

[4] Raskin J., Surmont V., Maat A.P.W.M. (2024). A randomized phase II study of extended pleurectomy/decortication preceded or followed by chemotherapy in patients with early-stage pleural mesothelioma: EORTC1205. Eur Respir J.

[5] Guzmán-Casta J., Carrasco-CaraChards S., Guzmán-Huesca J. (2021). Prognostic factors for progression-free and overall survival in malignant pleural mesothelioma. Thorac Cancer.

[6] Katz I., Straus C.M., Roshkovan L. (2023). Considerations for imaging of malignant pleural mesothelioma: a consensus statement from the International Mesothelioma Interest Group. J Thorac Oncol.

[7] Popat S., Baas P., Faivre-Finn C. (2022). Malignant pleural mesothelioma: ESMO clinical guidelines for diagnosis, treatment and follow up. Ann Oncol.

[8] Tsim S., Cowell G.W., Kidd A. (2020). A comparison between MRI and CT in the assessment of primary tumour volume in mesothelioma. Lung Cancer.

[9] Martini K., Meier A., Opitz I. (2016). Diagnostic accuracy of sequential co-registered PET+MR in comparison to PET/CT in local thoracic staging of malignant pleural mesothelioma. Lung Cancer.

[10] Schumann S.O., Kocher G., Minervini F. (2021). Epidemiology, diagnosis and treatment of the malignant pleural mesothelioma, a narrative review of the literature. J Thorac Dis.

[11] Opitz I., Scherpereel A., Berghmans T. (2020). ERS/ESTS/EACTS/ESTRO guidelines for the management of malignant pleural mesothelioma. Eur J Cardiothorac Surg.

[12] Patel A.M., Berger I., Wileyto E.P. (2017). The value of delayed phase enhanced imaging in malignant pleural mesothelioma. J Thorac Dis.

[13] Gill R.R., Umeoka S., Mamata H. (2010). Diffusion-weighted MRI of malignant pleural mesothelioma: Preliminary assessment of apparent diffusion coefficient in histologic subtypes. AJR Am J Roentgenol.

[14] Armato S.G., Coolen J., Nowak A.K. (2015). Imaging in pleural mesothelioma: a review of the 12th International Conference of the International Mesothelioma Interest Group. Lung Cancer.

[15] Opitz I., Friess M., Kestenholz P. (2015). A new prognostic score supporting treatment allocation for multimodality therapy for malignant pleural mesothelioma. J Thorac Oncol.

[16] Werner R., Caviezel C., Lauk O. (2020). Extended pleurectomy and decortication with resection and reconstruction of pericardium and hemidiaphragm for malignant pleural mesothelioma. J Vis Surg.

[17] Rusch V.W., Gill R., Mitchell A. (2016). A multicenter study of volumetric computed tomography for staging malignant pleural mesothelioma. Ann Thorac Surg.

[18] Rice D., Chansky K., Nowak A. (2016). The IASLC Mesothelioma Staging Project: proposals for revisions of the N descriptors in the forthcoming eighth edition of the TNM classification for pleural mesothelioma. J Thorac Oncol.

[19] Nowak A.K., Chansky K., Rice D.C. (2016). The IASLC mesothelioma staging project: proposals for revisions of the T descriptors in the forthcoming eighth edition of the TNM classification for pleural mesothelioma. J Thorac Oncol.

[20] Armato S.G., Francis R.J., Katz S.I. (2019). Imaging in pleural mesothelioma: a review of the 14th International Conference of the International Mesothelioma Interest Group. Lung Cancer.

[21] Rush V., Chansky K., Kindler H.L. (2016). The IASLC mesothelioma staging project: proposals for the M descriptors and for revision of the TNM stage groupings in the forthcoming (eighth) edition of the TNM classification for mesothelioma. J Thorac Oncol.

[22] Wolf A.S., Eisele M., Giroux D.J. (2024). The IASLC pleural mesothelioma staging project: expanded database to inform revisions in the Ninth edition of the TNM classification of pleural mesothelioma. J Thorac Oncol.

[23] Faccioli E., Terzi S., Giraudo C. (2022). Sarcopenia as a predictor of short- and long-term outcomes in patients surgically treated for malignant pleural mesothelioma. Cancers.

